# Genome wide identification and comparative analysis of glutathione transferases (GST) family genes in *Brassica napus*

**DOI:** 10.1038/s41598-019-45744-5

**Published:** 2019-06-24

**Authors:** Lijuan Wei, Yan Zhu, Ruiying Liu, Aoxiang Zhang, Meicheng Zhu, Wen Xu, Ai Lin, Kun Lu, Jiana Li

**Affiliations:** 1 Chongqing Engineering Research Center for Rapeseed, College of Agronomy and Biotechnology, Southwest University, Chongqing, 400716 China; 2 Academy of Agricultural Sciences, Southwest University, Chongqing, 400716 China

**Keywords:** Gene regulatory networks, Transcriptomics

## Abstract

Glutathione transferases (GSTs) are multifunctional enzymes that play important roles in plant development and responses to biotic and abiotic stress. However, a systematic analysis of GST family members in *Brassica napus* has not yet been reported. In this study, we identified 179 full-length *GST* genes in *B*. *napus*, 44.2% of which are clustered on various chromosomes. In addition, we identified 141 duplicated *GST* gene pairs in *B*. *napus*. Molecular evolutionary analysis showed that speciation and whole-genome triplication played important roles in the divergence of the *B*. *napus GST* duplicated genes. Transcriptome analysis of 21 tissues at different developmental stages showed that 47.6% of duplicated *GST* gene pairs have divergent expression patterns, perhaps due to structural divergence. We constructed a *GST* gene coexpression network with genes encoding various transcription factors (NAC, MYB, WRKY and bZIP) and identified six modules, including genes expressed during late seed development (after 40 days; *BnGSTU19*, *BnGSTU20* and *BnGSTZ1*) and in the seed coat (*BnGSTF6* and *BnGSTF12*), stamen and anther (*BnGSTF8*), root and stem (*BnGSTU21*), leaves and funiculus, as well as during the late stage of pericarp development (after 40 days; *BnGSTU12* and *BnGSTF2*) and in the radicle during seed germination (*BnGSTF14*, *BnGSTU1*, *BnGSTU28*, and *BnGSTZ1*). These findings lay the foundation for elucidating the roles of GSTs in *B*. *napus*.

## Introduction

Glutathione transferases (GSTs) are multifunctional enzymes that are widely distributed in various organisms. GSTs contain an N-terminal domain and a C-terminal domain. The N-terminal domain includes a catalytic residue for glutathione (GSH) binding and catalysis, whereas the less conserved C-terminal domain, comprising five or six major helices, binds hydrophobic substrates and determines GST specificity and activity^1^. GSTs are divided into 14 classes: phi (GSTF), tau (GSTU), theta (GSTT), zeta (GSTZ), lambda (GSTL), metaxin, hemerythrin (GSTH), iota (GSTI), glutathione-dependent dehydroascorbate reductase (DHAR), tetrachloro hydroquinone dehalogenase (TCHQD), γ-subunit classes of the eukaryotic translation elongation factor 1B (EF1Bγ), glutathionyl hydroquinone reductase (GHR), GSTs with two thioredoxins (GST2N), and microsomal prostaglandin E synthase type 2 (mPGES2)^2,3^. The phi and tau classes are the most abundant classes in plants. The GSTL and DHAR classes are monomeric, with no GSH-conjugating activity towards xenobiotic substrates and function in redox homeostasis^4,5^. GSTI has been identified only in non-vascular plants, algae and cyanobacteria, and appears to have been lost during the evolution of most terrestrial plants^3,6^.

GSTs were initially found to function in herbicide detoxification^7^. Their xenobiotic detoxification function can be detected using the 1-chloro-2,4-dinitrobenzene (CDNB) assay, in which the chloro group of CDNB is substituted by glutathione^2^. GSTs catalyze the conjunction of electrophilic substrates with the tripeptide GSH. Chronopoulou *et al*. (2017) reviewed the roles of GSTs based on recent progress in plant proteomics, genomics and transcriptomics analyses. In addition to their catalytic activities, GSTs function as non-catalytic proteins by binding to flavonoids and anthocyanins and transporting them from the cytoplasm into the central vacuole^8–10^. GSTs can bind to the phytohormones auxin^11^ and cytokinin^12^, which are involved in plant development. GSTs can also bind to porphyrinogens^13^ and oxylipins^14^, thereby protecting plant cells from oxidative stress. In addition, plant GSTs play important roles in responses to biotic and abiotic stresses, such as pathogen attack^15,16^, heavy metals^17^, drought^18^ and salt^19^, as well as salicylic acid signaling^16^. However, the detailed mechanisms are currently unclear.

*Brassica napus* is an allopolyploid species that formed via hybridization of the diploid species *B*. *oleracea* and *B*. *rapa*. The model dicot *Arabidopsis thaliana* has been reported to contain 55 GSTs in eight classes, although the EF1Bγ, GHR, metaxin, GSTH and GST2N classes were not included in the analysis^2^, whereas there are 101 GSTs in soybean^20^, 81 in poplar^21^, 42 in potato^22^ and 99 in sorghum^23^, 75 in *B*. *rapa*^24^ and 65 in *B*. *oleracea*^25^. The duplicated GST genes in soybean were formed by whole-genome duplication (WGD)^19^, whereas the expansion of tau and phi GSTs in *Capsella rubella* mainly occurred through tandem gene duplication^26^. In *B*. *rapa*, WGD and tandem duplication played the main role in the expansion of GSTs^23^. However, to date, a systematic analysis of the GST family in *B*. *napus* has not been reported. The availability of whole-genome sequences for *B*. *rapa*^27^, *B*. *oleracea*^28^, and *B*. *napus*^29^ provides valuable resources for studying the GST family in *Brassica* species. In this study, we identified GSTs of all 13 classes in *B*. *napus* and analyzed their evolution and syntenic relationships. We also evaluated their expression patterns in different tissues and in response to biotic stresses. Our results shed light on this important gene family in the crop *B*. *napus*.

## Materials and Methods

### Identification and nomenclature of GSTs in *B*. *napus*

We identified GSTs from all 13 classes for analysis. The resulting sequences for 66 proteins from *A*. *thaliana* (http://www.arabidopsis.org/)^2^, 59 from rice (http://rice.plantbiology.msu.edu/)^30^, 81 from *Populus trichocarpa* (https://phytozome.jgi.doe.gov/)^21^, 37 from *Physcomitrella patens* (http://plants.ensembl.org/index.html)^3^, and 575 from various animals, fungi, and bacteria retrieved from Lan *et al*.^21^ were obtained (Supplementary Table 1) and used as query sequences to identify GST proteins in *B*. *napus* v5.0 (http://www.genoscope.cns.fr/brassicanapus/)^29^ by BLASTP analysis, with an e-value of 1e-10. In addition, GST_N and GST_C domains were identified by searching the Pfam database (http://Pfam.sanger.ac.uk/) with e-value of 1.0 and the conserved domain database (CDD) at NCBI (https://www.ncbi.nlm.nih.gov/Structure/cdd). GSTs without conserved domains were excluded from the analysis. For nomenclature, the prefix ‘*Bn*’ for *B*. *napus* was used, followed by GST and a unique number, such as *BnGST1*. Gene structures and motifs were analyzed using TBtools (https://github.com/CJ-Chen/TBtools)^31^. To explore the evolution of the *GSTs* in *Brassica*, the *GST* sequences from *B*. *rapa* v 1.5^27^ and *B*. *oleracea* v2.1 (http://plants.ensembl.org/index.html) were obtained as described above.

### Phylogeny of the *GSTs*

GST protein sequences from *B*. *napus* were aligned using MAFFT version 7^32^ with default parameters, and phylogenic trees were constructed using the maximum likelihood method using PHYML 3.0 with the Jones, Taylor and Thornton model with 100 bootstrap replicates^33^. FigTree v1.4.3 (http://tree.bio.ed.ac.uk/software/figtree/) was used to visualize the phylogenetic trees.

### Distribution of *GST* genes, gene duplication, molecular evolutionary and Pearson correlation analysis

The distribution of *GST* genes on *B*. *napus* chromosomes was displayed using MapChart version 3.0. The *GSTs* were clustered together on the chromosomes: two or more *GSTs* separated by no more than three genes were referred to as a *GST* gene cluster. To identify the form of gene duplication, 101,040 *B*. *napus* gene sequences were aligned using BLASTp, with an e-value of 1e-10. MCScanX with default values was used to classify the duplication patterns of the GSTs into segmental, tandem, proximal, and dispersed duplications^34^. In addition, syntenic blocks with at least ten genes in the *Brassica* genome were identified. Duplicate gene pairs were identified according to the following criteria^35^: candidate duplicate gene pairs were located in syntenic blocks, and duplicate gene pairs were grouped together in the phylogenetic tree. KaKs_Calculator 2.0^36^ was used to calculate the ratio of the nonsynonymous substitution rate (Ka) to the synonymous substitution rate (Ks), and the ω (Ka/Ks) value between paralogous gene pairs was determined using the MYN (Modified YN) model. Divergence time was inferred using the formula T = Ks/2R, where R is 1.5 × 10^−8^ synonymous substitutions per site per year^37^. The Pearson correlation coefficients among duplicated gene pairs were calculate using the cor command in R.

### Promoter analysis

The promoter sequences in the regions 1500 bp upstream of the coding sequences were obtained, and the *cis*-acting elements were analyzed using the PlantCARE website (http://bioinformatics.psb.ugent.be/webtools/plantcare/html/)^38^ and the PLACE database^39^.

### Expression analysis of *BnGST*s during development and in response to biotic stresses in *B*. *napus*

Transcriptome sequencing was performed using 21 different *B*. *napu*s tissues (root, stem, leaves, bud, funiculus, anthocaulus, anther, calyx, capillament, petal, stamen, pistil, shoot apex, silique pericarp, seed, seed coat, cotyledon, episperm, endopleura and embryo) at different stages of development; the sequencing datasets were deposited in NCBI under BioProject ID PRJCA001246. Sequencing libraries were generated using an Illumina RNA Library Prep Kit following the manufacturer’s recommendations. Sequencing reads were aligned to the *B*. *napus* reference genome and assembled using TopHat 2.0.0 and Cufflinks with default parameters^40^. Gene expression levels were estimated using FPKM (fragments per kilobase of exon per million mapped fragments). *BnGST* expression levels were obtained in response to treatment with *Sclerotinia sclerotiorum*^41^ and *Leptosphaeria maculans*^42^. A heatmap was generated using the R package pheatmap.

### Weighted gene coexpression network analysis (WGCNA)

A gene coexpression network was constructed using the WGCNA package in R^43^. The expression levels in different tissues were log-transformed via log_2_ (FPKM + 1), and genes with low expression levels (maximum log_2_ (FPKM + 1) <4) were filtered out. Genes involved in responses to pathogens were also used to construct the network. The settings used were as follows: minModuleSize = 30, maxBlockSize = 6000, reassignThreshold = 0, mergeCutHeight = 0.25, TOMType = “unsigned”. Genes with WGCNA edge weight >0.1 were displayed using Cytoscape 3.6.1^44^.

## Results

### Identification of GSTs in *B*. *napus*

**Figure 1:**
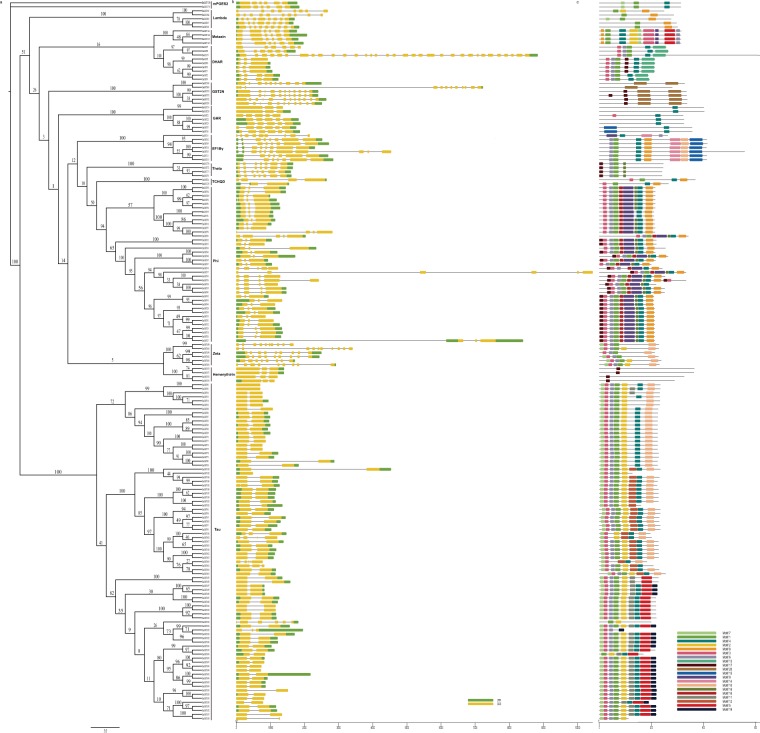
Phylogenetic analysis (**a**), gene structures (**b**) and gene motifs (**c**) of *GSTs* in *B*. *napus*.

To identify GST proteins in various *Brassica* species, we performed BLASTP with an e-value of 1e-10. After searching for conserved GST-N and GST-C domains using the Pfam database and CDD at NCBI, we identified 179 full-length genes encoding GST proteins in *B*. *napus* (Supplementary Table 2). To classify the *B*. *napus* GSTs, we examined their phylogenetic relationships using the maximum likelihood method. Based on the maximum likelihood tree, the 179 GSTs were divided into 13 classes, including mPGES2, GST2N, hemerythrin (GSTH), zeta (GSTZ), EF1Bγ, theta (GSTT), TCHQD, DHAR, GSTF, metaxin (MTX), lambda (GSTL), GHR and tau (GSTU) (Fig. 1a). The phi and tau classes were the largest, with 39 and 84 members, respectively. mPGES2, GST2N, GSTH, GSTZ, EF1Bγ, GSTT, TCHQD, DHAR, MTX, GSTL, and GHR included 2, 6, 4, 6, 7, 4, 2, 9, 4, 5, and 7 members, respectively. We also identified 85 *GST* genes in *B*. *oleracea* and *B*. *rapa* (Supplementary Table 2). The phi and tau classes of GSTs in *B*. *oleracea* and *B*. *rapa* were also the largest, accounting for 72.6% and 70.6% of all GSTs, respectively. The proportion of all *GST* genes in *B*. *napus*, *B*. *oleracea*, and *B*. *rapa* were 0.21%, 0.18%, and 0.15%, respectively. There was no correlation between genome size and the number of *GST* genes.

The gene structures and positions of these GSTs were conserved (Fig. 1b). Most (76.9%) phi class *GSTs* had three exons, and 73 of the 82 tau (89.0%) *GSTs* had two exons. GST2N, zeta and lambda *GSTs* had approximately 10 exons, while EF1Bγ and theta *GSTs* had seven exons, and metaxin GSTs had six exons. The gene motifs of the *GSTs* were also conserved (Fig. 1c).

In addition to the full-length GSTs, 20, 11, and 7 GST fragments containing partial N- and C-domains were found in *B*. *napus*, *B*. *oleracea*, and *B*. *rapa*, respectively (Supplementary Table 3). The fragments encoded putative polypeptides ranging from 38 to 418 amino acids. We considered these *GST* fragments to be pseudogenes.

### Chromosomal distribution

**Figure 2:**
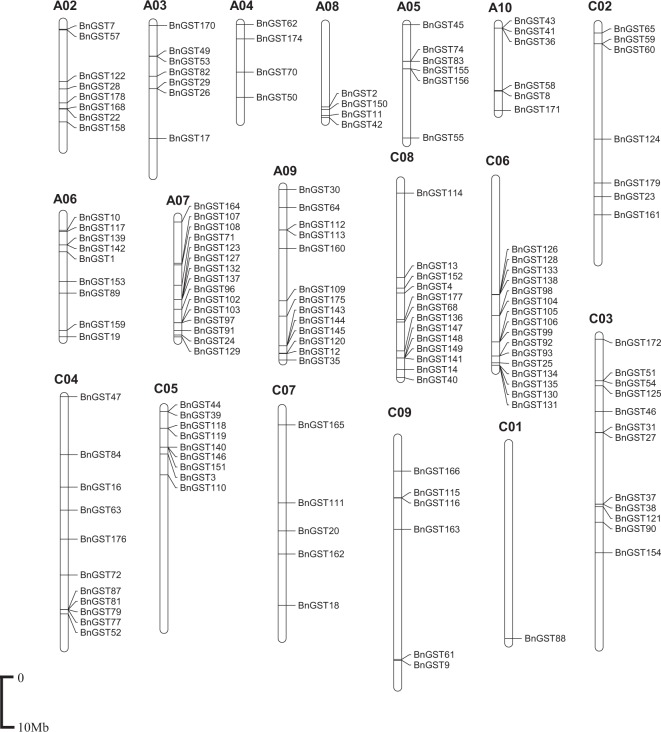
Chromosomal distribution of *GSTs* in *B*. *napus*.

We investigated the chromosomal distribution of *GST* genes in the *Brassica* species. In total, 152 of the 179 full-length *GSTs* were distributed on all 19 *B*. *napus* chromosomes except chromosome A1 (72 on the An genome and 80 on the Cn genome), while the 27 other *GSTs* were assigned to random chromosomes (13 on the An genome and 14 on the Cn genome) (Fig. 2). Full-length *B*. *oleracea* and *B*. *rapa GSTs* were found on all chromosomes. The *GST* fragments were found on 8 of the 19 *B*. *napus* chromosomes, 6 of the 9 *B*. *oleracea* chromosomes and 4 of the 10 *B*. *rapa* chromosomes. Notably, these *GSTs* are unevenly distributed on the chromosomes. High-density regions harboring GSTs were discovered on chromosome A7, A9, C3, C4, C5, C6 and C8 in *B*. *napus* (Fig. 2). In *B*. *oleracea*, chromosomes C4 and C6 contained the most *GST* genes (15), whereas C1 contained only one *GST*. In *B*. *rapa*, chromosome A7 and A9 contained the most *GST* genes, whereas A1 contained only one *GST* (Supplementary Table 2).

In total, 31 *GST* clusters containing 88 *GSTs* were found on 13 of the 19 *B*. *napus* chromosomes, representing 44.2% of *GST* genes. Chromosome C6, containing 15 *GST* genes, harbored the most clusters (5). Most of the clusters contained two genes, whereas cluster 18 on chromosome C5 contained six genes, and cluster 7 on A7 and cluster 12 on A10 each contained five genes. Finally, 15 clusters containing 43 *GSTs* (44.8%) and 12 clusters containing 33 *GSTs* (35.9%) were found in *B*. *oleracea* and *B*. *rapa*, respectively.

### Phylogenetic and synteny analysis of GSTs in *A*. *thaliana*, *B*. *rapa*, *B*. *oleracea*, and *B*. *napus*

We constructed a phylogenetic tree based on the 66 *A*. *thaliana*, 85 *B*. *rapa*, 85 *B*. *oleracea*, and 179 *B*. *napus* GSTs (Supplementary Fig. 1). Metaxin and mPGES2 appeared to be ancient classes of GSTs. Indeed, metaxins have previously been reported to be an ancient GST superfamily^45^. The mPGES2 GSTs, comprising two members, were evolutionarily distant from the other groups^2^. The remaining GST proteins were divided into 11 classes: GSTF, GSTT, EF1Bγ, GSTZ, DHAR, TCHQD, GST2N, GHR, GSTL, GSTH, and GSTU. The tau class of GSTs was the largest, followed by the phi class. AtGSTF2 had the most orthologs in *Brassica*, including ten in *B*. *napus*, five in *B*. *rapa*, and five in *B*. *oleracea*.

**Figure 3:**
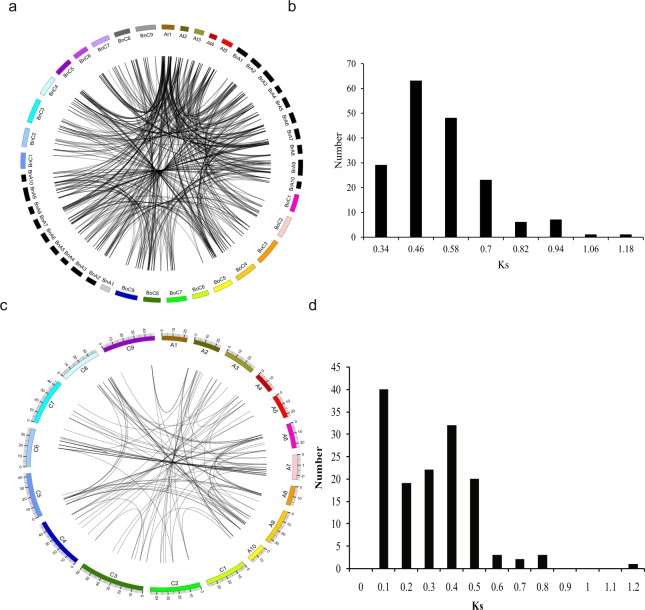
Duplicated and orthologous genes in *Brassica*. (**a**) circle plot of orthologous genes in *A*. *thaliana* (At), *B*. *oleracea* (Bo), *B*. *rapa* (Br), and *B*. *napus* (Bn); (**b**) density of Ks values of *GST* gene pairs between *B*. *napus* and *A*. *thaliana*; (**c**) duplicate gene pairs in *B*. *napus*; (**d**) density of Ks values of duplicated *GST* genes in *B*. *napus*.

To explore the evolution of the *GSTs* in *Brassica*, we constructed synteny maps of *GSTs* in *A*. *thaliana*, *B*. *oleracea*, *B*. *rapa*, and *B*. *napus* (Fig. 3a) and determined the retention or loss patterns of orthologous genes based on their syntenic relationships. We detected orthologs for 55 *A*. *thaliana GST* genes in *B*. *napus*, *B*. *rapa*, and *B*. *oleracea*. Based on the syntenic relationship between *A*. *thaliana* and *B*. *napus*, a total of 163 collinear gene pairs were identified, including 6, 22, 7, 13, 2, 2, 1 and 1 *GST* gene in *A*. *thaliana* with 1, 2, 3, 4, 5, 6, 8 and 10 orthologs in *B*. *napus*, respectively (Supplementary Table 4). In addition, we identified 61 collinear gene pairs between *A*. *thaliana* and *B*. *oleracea*: 26, 10, 2, 1, and 1 *GST* genes in *A*. *thaliana* have 1, 2, 3, 4, and 5 orthologs in *B*. *oleracea*, respectively. In total, 79 collinear gene pairs were found between *A*. *thaliana* and *B*. *rapa*: 25, 15, 5, 1, and 1 *A*. *thaliana* gene has 1, 2, 3, 4, and 5 orthologs in *B*. *rapa*, respectively.

Homeologous gene pairs in *B*. *napus* were identified based on high sequence similarity of pairs of genes and location on homeologous chromosomes. These 199 *GSTs* in *B*. *napus* (179 full-length GSTs and 20 GST fragments) were found as 74 homeologous gene pairs on homeologous chromosomes (Supplementary Table 4). According to the synteny analysis in *B*. *napus* and its diploid progenitors *B*. *oleracea* and *B*. *rapa*, a total of 79 gene pairs maintained their relative positions between the An subgenome of *B*. *napus* and the Ar genome of *B*. *rapa*, and 62 gene pairs maintained their relative positions between the Cn subgenome of *B*. *napus* and Co genome of *B*. *oleracea*.

### The expansion of *GST* genes in *B*. *napus*

To understand the mechanism underlying the expansion of the *GST* family in *B*. *napus*, we examined the types of *GST* gene duplication. Of the 101,040 genes in the *B*. *napus* genome, 3422 genes (3.4%) appeared to have undergone tandem duplication and 61,310 (60.7%) genes had undergone segmental duplication (Supplementary Table 5). We found that 146 of 199 *GSTs* (73.4%, *P* > 0.05%) were derived from segmental duplication, a number slightly larger than the average percentage at the whole-genome level (60.7%). Therefore, it appears that segmental duplication played an important role in the expansion of the *GST* family in *B*. *napus*. In addition, we examined the *GST* gene expansion patterns in *B*. *oleracea* and *B*. *rapa*, finding that most *BoGSTs* (56.2%; 54/96) and *BrGSTs* (63.0%;58/92) were derived from segmental duplication, followed by tandem duplication (19.8% in *B*. *oleracea* and 21.7% in *B*. *rapa*) (Supplementary Table 5).

The orthologous GST gene pairs between *B*. *napus* and *A*. *thaliana* were used to estimate the Ks value. The Ks values for all orthologous gene pairs ranged from 0.2266 to 1.0967, with an average of 0.4857 (Fig. 3b). The divergence time ranged from 7.55 MYA to 36.56 MYA, with an average value of 15.89 MYA. These results indicated that GSTs of *B*. *napus* diverged from *A*. *thaliana* ~16 MYA, which was consistent with the recent whole-genome triplication event that occurred approximately 9–15 MYA, or even 28 MYA^46^.

Among the *GSTs* in the three *Brassica* species examined, we identified 141 duplicate gene pairs in *B*. *napus* (Fig. 3c). We estimated the timing of the whole-genome duplication (WGD) event based on the distribution of Ks values, which ranged from 0.0064 to 1.1531 and averaged 0.2612. The corresponding duplication time ranged from 0.21 to 38.44 MYA, with an average value of 8.7 MYA (Supplementary Table 5). Two peaks of Ks values were observed in *B*. *napus*: one peak (0–0.1) represented the duplication time of these genes, which occurred during the formation of *B*. *napus* 7500–12,500 years ago (Fig. 3d), and the other peak (0.3–0.4), representing a duplication time of ~10 MYA, corresponded to the *Brassica* whole-genome triplication event (9–15 MYA). Therefore, the processes of *B*. *napus* speciation and *Brassica* whole-genome triplication likely played important roles in the divergence of the *GST* duplicated genes in *B*. *napus*.

Ka/Ks value <1 indicates that a gene pair has experienced negative selection, whereas Ka/Ks >1 indicates positive selection and Ka/Ks = 1 indicates neutral selection. The Ka/Ks ratios for most GST collinear gene pairs were <1, except for the gene pair *BnGST129* and *BnaGST139* (Ka/K >1). These results indicated that most genes have experienced negative selection, whereas *BnGST129* and *BnGST139* experienced positive selection (Supplementary Table 6).

### Extensive changes in exon–intron structure between duplicate gene pairs

We identified 141 duplicate gene pairs, 131 of which contained full-length GSTs in each duplicate. Of the 131 full-length duplicate gene pairs, 28 pairs (21.4%) showed different numbers of exons (Supplementary Table 6). In 45 other duplicates (34.3%), the number of exons was the same, but the lengths of one or more exons differed. Thus, 55.7% of the duplicated genes exhibited obvious structural divergence.

### Identification of *cis*-acting elements in *GST* genes

We next characterized the *cis*-acting elements in promoter regions of the *BnGST* genes. Various abiotic- and biotic-stress related *cis*-elements were identified. The abiotic-stress-related *cis*-acting elements included light-responsive elements (G-box and I-box), abscisic acid-responsive elements (ABRE, ACGT box), GA-responsive element (GARE), auxin-responsive element (AuxRE), dehydration-responsive element (DRE), ethylene-responsive element (ERE), heat-responsive element (HSE), low temperature-responsive element (LTRE), and sugar-response element (SRE). GSTs might also be involved in responses to ammonium, copper, sulfur, and phosphate. We also identified biotic-stress-related *cis*-elements, such as ethylene- and pathogen-responsive element (GCC-box), and wounding- and pathogen-responsive element (W box). In addition, there were various tissue-specific *cis*-regulatory elements, including the seed-specific (embryo and endosperm) element AACA motif, the floral organ element CArG and the xylem-specific element AC box. Finally, many predicted transcription factor (TF) binding sites were found, such as NAC, MYB, WRKY and bZIP binding sites (Supplementary Table 7).

### Expression of *GSTs* in different *B*. *napus* tissues

**Figure 4:**
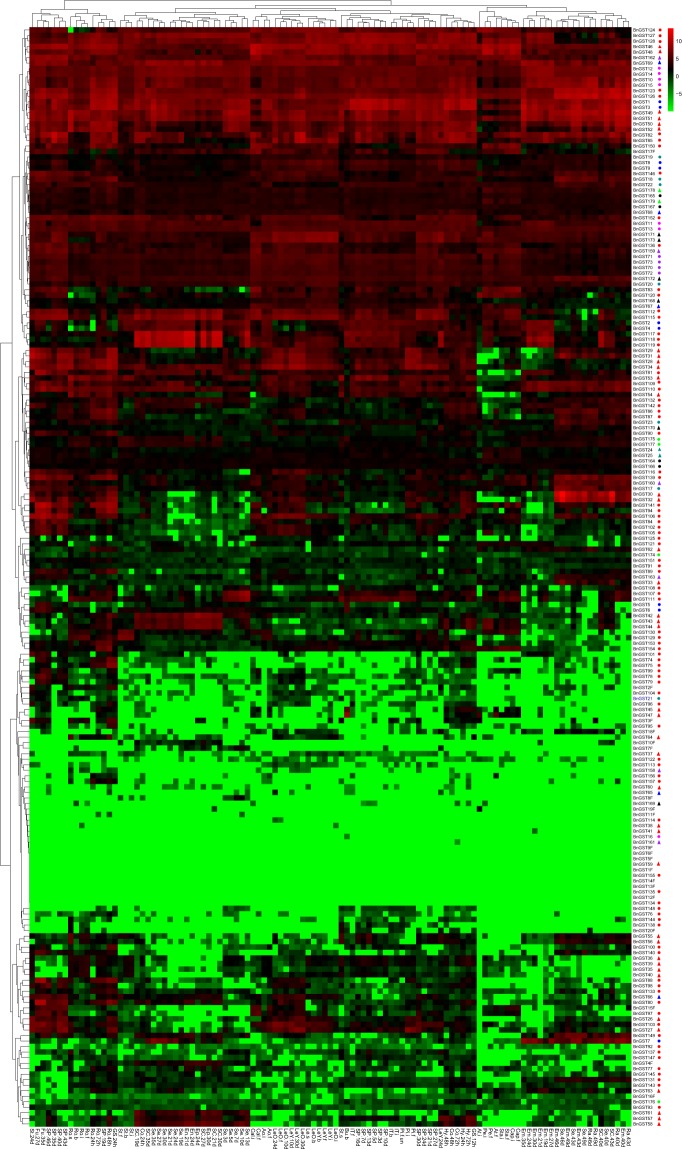
Expression patterns of all 199 *GSTs* in 21 different tissues at different developmental stages in *B*. *napus*. The color bar represents log_2_ expression levels (FPKM). The group information for *B*. *napus* GSTs is indicated: red, blue, magenta, dark cyan, purple, black, and green circles represent tau, DHAR, EF1Bγ, GHR, theta, metaxin, hemerythin class GSTs; red, blue, dark cyan, purple, black, green triangles represent phi, lambda, TCHQD, zeta, GST2N and mPEGS2 class GSTs.

To explore the expression patterns of *GSTs* in *B*. *napus*, we analyzed their expression levels in 21 different tissues at different developmental stages, including root, stem, leaf, bud, funiculus, anthocaulus, anther, calyx, capillament, petal, stamen, pistil, shoot apex, silique pericarp, seed, seed coat, cotyledon, episperm, endopleura, and embryo tissue. Of the 199 *GSTs*, 58 were expressed in all tissues at different developmental stages, whereas 51 *GSTs*, including 16 *GST* fragments, exhibited almost no expression (Fig. 4). The 90 remaining *GSTs* were expressed in specific tissues. Most *GST* fragments were not expressed any tissues and were excluded from further expression analysis. Several genes were expressed in all tissues except petals, capillaments, stamens, and anthers, including *BnGST28*, *BnGST29*, *BnGST31* and *BnGST34* (*GSTF3*), *BnGST53* and *BnGST54* (*GSTF10*), *BnGST81* (*GSTU4*), *BnGST86* and *BnGST87* (*GSTU7*), *BnGST109* and *BnGST110* (*GSTU13*), *BnGST132* (*GSTU22*), and *BnGST142* (*GSTU25*). *BnGST57*, *BnGST58* and *BnGST61* (*GSTF12*) were expressed during early seed development and seed coat formation (before day 40), while *BnGST149* (*GSTU25*) was expressed during all stages of seed development and seed coat formation.

Of the 131 full-length *GST* duplicate pairs, five were almost undetectable in any tissue, and the remaining 126 showed expression in some or all tissues. The Pearson correlation coefficient (with a cutoff of 0.6) was used to investigate expression patterns of duplicated GST gene pairs. In total, 39 of the 126 (30.9%) were expressed in all tissues, and the correlation coefficients for the expression levels of 25 duplicated gene pairs were more than 0.6, which showed that the expression patterns of these gene pairs were similar (Supplementary Table 6). The 77 (61.1%) remaining duplicate *GST* pairs showed different expression patterns, which were classified into four groups: (I) one copy of each duplicate was expressed in all tissues and the other was not expressed in any tissue; (II) one copy was expressed in all tissues and the other was expressed in a specific tissue; (III) one copy was expressed in a specific tissue and the other was not expressed in any tissue; and (IV) both duplicates were expressed in specific tissues. Group I contained 7 *GST* pairs, group II contained 10, group III contained 17, and group IV contained 53 *GST* pairs. The expression pattern of all duplicated gene pairs in group I, 8 of 10 duplication gene pairs in group II, 16 of 17 in group III, and 15 of 53 in group IV were different. In total, 60 of 126 expressed GST duplicated gene pairs (47.6%) had different expression patterns, indicating that these duplicated *GST* genes have undergone rapid divergence (Supplementary Table 6). In addition, 37 of 60 (61.7%) gene pairs showed structural divergence.

Of the 31 *GST* clusters, 29 contained full-length *GSTs*: Clusters 6 and 9 contained fragments and were excluded from expression analysis. Marked divergence in gene expression was also observed among *GST* family members (Supplementary Fig. 2). *GSTs* in Cluster 4, 11, 13, and 16 were not expressed in any tissue. *BnGST49* in Cluster 1 and *BnGST51* in Cluster 14 were expressed in all tissues, while *BnGST53* in Cluster 1 and *BnGST54* in Cluster 14 were expressed in all tissues except petal, capillament, stamen, and anther tissue. Some *GSTs* in Cluster3, 10, 20, 21 and 30 were expressed in all tissues, while other *GSTs* were barely expressed in any tissue. Among Cluster 15 genes, *BnGST31* (*GSTF3*) was not expressed in petal, capillament, stamen, or anther tissue, while *BnGST27* (*GSTF2*) showed little or no expression during seed development in tissues such as seed, seed coat, episperm, endopleura, cotyledon, and embryo in addition to petal, capillament, stamen, and anther tissue.

### Construction of an expression network

**Figure 5:**
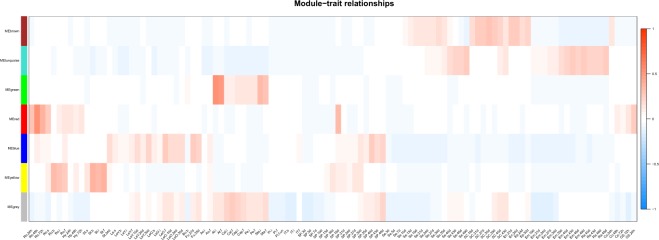
Module-trait relationships detected by GST and transcription factor (NAC, MYB, WRKY and bZIP) coexpression networks in *B*. *napus*. Each row represents a module and each column represents a tissue. The color scale shows the correlation between each module and trait from −1 (blue) to 1 (red). GS, germinate seed; Ro, root; Hy, hypocotyl; St, stem; Ao, anthocaulus; Le, leaf; Bu, bud; Fu, fulicus; Ao, anthocaulus; At, anther; IT, shoot apex; Cal, calyx; Cap, capillament; Pe, petal; Sta, stamen; Pi, pistil; SP, silique; Se, seed; SC, seed coat; Em, embryo; En, endopleura; Ep, episperm; Ra; Co, cotyledon. For stages, s, seedling stage; b, bud stage; i, initial flowering stage; and f, full-bloom stage. The time after seed germination is indicated as 24, 48, and 72 h. The number of days after pollination (DAP) is indicated as 3, 5, 7, 10, 13, 19, 21, 30, 40, and 46 d.

Constructing coexpression networks is an effective way to identify clusters of genes with similar functions. In the current study, no module was identified when we analyzed *GSTs* expressed in tissues at different developmental stages, revealing the divergent functions of GSTs. TFs including NAC, MYB, WRKY and bZIP TFs are thought to regulate the expression of *GSTs*. To compare the expression patterns of the genes for these TFs and *GSTs*, six modules were identified using WGCNA with a power of 10. Genes in the turquoise module were expressed during the late stage of seed development (after 40 days), including embryo and radical development. For example, *BnGST7* (*DHAR3*), *BnGST123* and *BnGST126* (*GSTU19*), *BnGST128* (*GSTU20*), *BnGST131* (*GSTU21*), and *BnGST160* (*GSTZ1*) shared similar expression patterns with the TF genes *NAC32*, *NAC60*, *NAC89*, *WRKY32*, and *ABI5*. *BnGST10* and *BnGST15* (*EF1Bγ1*) shared similar expression patterns with *WRKY2* and *MYB3* (Supplementary Table 8, Fig. 5, Table 1). Genes in the brown module, which are involved in seed coat development, including *BnGST42*, *BnGST43*, and *BnGST44* (*GSTF6*), *BnGST58* and *BnGST61* (*GSTF12*) and *BnGST117* (*GSTU17*), were coexpressed with *MYB5*, *MYB56*, *MYB61*, *MYB118*, *TTG2*, and *TT2*. *BnGST47* (*GSTF8*), the gene in the green module, which is involved in stamen and anther development, was coexpressed with *MYB3*, *MYB21*, *MYB101*, *MYB108*, *MYB57*, *MYB78*, and *MYB117*. *BnGST129* (GSTU21), the gene in the yellow module, was involved in root and stem development and was coexpressed with *MYB103*, *MYB46*, *MYB69*, *MYB85*, *NAC73*, *NAC12*, and *NAC66*. Genes in the blue module were primarily expressed in leaves, the funiculus and during the late stage of pericarp development (after 40 days). Most of these *GSTs* were coexpressed with WRKY TFs. For example, *BnGST102*, *BnGST103*, and *BnGST105* (*GSTU12*) and *BnGST27* (*GSTF2*) were coexpressed with *WRKY25*, *WRKY26*, and *WRKY33*. In addition, the expression patterns of *BnGST168* and *BnGST171* (GST_2_N) and *BnGST173* (*GSTH1*) were similar to that of *NAC35*. Finally, genes in the red module, which are expressed in the radical development during seed germination, including *BnGST64* (*GSTF14*), *BnGST74* and *BnGST75* (*GSTU1*), *BnGST156* (*GSTU28*), and *BnGST157* (*GSTZ1*) shared similar expression patterns with *WRKY34*, *WRKY36*, *WRKY40*, *WRKY65*, and *WRKY72*.

**Table 1:** Coexpressed GST and transcription factor (NAC, MYB, WRKY and bZIP) genes in various in *B*. *napus* tissues.

Module	*GST* Genes	Tissue	TF Genes
Turquoise	*BnGST7* (*DHAR3*), *BnGST123* and *BnGST126* (*GSTU19*), *BnGST128* (*GSTU20*), *BnGST131* (*GSTU21*) and *BnGST160* (*GSTZ1*)	Seed	*NAC32*, *NAC60*, *NAC89*, *WRKY32*, *ABI5*
	*BnGST10*, *BnGST15* (*EF1Bγ1*)	Seed	*WRKY2* and *MYB3*
Brown	*BnGST42*, *BnGST43* and *BnGST44* (*GSTF6*), *BnGST58* and *BnGST61* (*GSTF12*), *BnGST117* (*GSTU17*)	Seed coat	*MYB5*, *MYB56*, *MYB61*, *MYB118*, *TTG2* and *TT2*
Green	*BnGST47* (*GSTF8*)	Stamen and anther	*MYB3*, *MYB21*, *MYB101*, *MYB108*, *MYB57*, *MYB78*, *MYB117*
Yellow	*BnGST129* (*GSTU21*)	Root and stem	*MYB103*, *MYB46*, *MYB69*, *MYB85*, *NAC73*, *NAC12*, *NAC66*
Blue	*BnGST102*, *BnGST103* and *BnGST105* (*GSTU12*), *BnGST27* (*GSTF2*)	Leaf, funiculus and late-stage pericarp Leaf, funiculus and late-stage pericarp	*WRKY25*, *WRKY26*, *WRKY33*
	*BnGST168* and *BnGST171* (*GST_2_N*), *BnGST173* (*GSTH1*)		*NAC35*
Red	*BnGST64* (*GSTF14*), *BnGST74* and *BnGST75* (*GSTU1*), *BnGST156* (*GSTU28*), *BnGST157* (*GSTZ1*)	Radicle during seed germination	*WRKY34*, *WRKY36*, *WRKY40*, *WRKY65*, *WRKY72*

We also analyzed the expression patterns of 99 *GST* duplicated pairs (log_2_ (FPKM + 1) >4) using WGCNA. In total, 49 of 97 (50.5%) *GST* duplicated gene pairs were in different modules, which showed that these duplicated gene pairs likely had different functions (Supplementary Table 8). From these results and the correlation analysis of expression levels of GST duplicated gene pairs above, we conclude that *GST* duplicated gene pairs have divergent roles in the growth and development of *B*. *napus*.

### Expression of *GSTs* in response to biotic stress

Blackleg disease (caused by *L*. *maculans*) and white stem rot (caused by *S*. *sclerotiorum*) are the most serious diseases of *B*. *napus*. We therefore examined the expression patterns of the *GSTs* in response to these pathogens (Supplementary Figs 3 and 4). The most pathogen-responsive genes included *BnGST101* and *BnGST102* (*GSTU12*), *BnGST26* (*GSTF2*), *BnGST29* (*GSTF3*), *BnGST66* (*GSTL1*), *BnGST81* (*GSTU4*), *BnGST88* (*GSTU8*), *BnGST99* (*GSTU11*), *BnGST110* (*GSTU13*), *BnGST139* (*GSTU24*), and *BnGST141* (*GSTU25*). In addition, *BnGST44* (*GSTF6*), *BnGST58*, and *BnGST61* (*GSTF12*) appeared to be important for resistance to *L*. *maculans*.

## Discussion

### Duplication and evolution of GST genes in *Brassica*

In our study, we identified 179, 85 and 85 full-length *GST* genes in *B*. *napus*, *B*. *oleracea* and *B*. *rapa*, respectively. Khan *et al*. (2018) identified 75 GSTs in eight classes for *B*. *rapa*, but did not include the mPEGS2, GHR, metaxin, GSTH and GST2N classes^24^. Vijayakumar *et al*. (2016) found 65 *B*. *oleracea* GSTs that were divided into 11 classes, but did not include the metaxin and GSTH classes^25^. We have included all 13 GST classes in our analyses, so the numbers of GSTs in *B*. *oleracea* and *B*. *rapa* were higher than those in previous studies. Whole-genome triplication (WGT) has occurred in *Brassica* species^46^. Given that there were 66 GST genes in *A*. *thaliana*, after WGT more than 198 GST genes should be found in *B*. *rapa* and *B*. *oleracea*. In our study, 85 BrGSTs and 85 BoGSTs were found, which indicates that duplicated genes might have been lost after WGT. About 35% genes have been lost via deletion after the divergence of *A*. *thaliana* and *Brassica*^47^. In total 79 collinear gene pairs were found between *A*. *thaliana* and *B*. *rapa*, whereas 61 collinear gene pairs were found between *A*. *thaliana* and *B*. *oleracea*, perhaps due to assembly errors in the currently available *B*. *oleracea* genome information. With the release of new *B*. *rapa* and *B*. *oleracea* reference genomes based on Nanopore technology^48^, we should be able to detect the retention or loss of gene families more accurately to avoid the false observation due to the incompleteness of the reference genome or annotation errors.

B. *napus*, an allopolyploid, was formed by hybridization between the diploid progenitors *B*. *rapa* and *B*. *oleracea* (U 1935). The number of GSTs (179) in *B*. *napus* was roughly equal to the sum of *B*. *rapa* (85) and *B*. *oleracea* (85). The synteny analysis between *B*. *napus* and its diploid progenitors *B*. *oleracea* and *B*. *rapa* indicated that most GST genes in *Brassica* were located in the syntenic regions, with 79 gene pairs shared between the An subgenome of *B*. *napus* and the Ar genome of *B*. *rapa*, and 62 gene pairs shared between the Cn subgenome of *B*. *napus* and the Co genome of *B*. *oleracea*. However, we still find that some GST genes in diploid progenitors were lost. The progenitor species had different numbers of chromosomes, and the A and C subgenomes have undergone rearrangements; thus, genes could be lost in the process of polyploidization.

### *GSTF6* and *GSTF12* are essential for seed coat development

In the current study, coexpression network analysis suggested that *BnGSTF6* and *BnGSTF12* are involved in seed coat development. Indeed, *GSTF12* has been shown to play important roles in seed and fruit coloration in plants. In *Arabidopsis*, AtGSTF12 (TT19) functions as a carrier that transports anthocyanin from the cytosol to tonoplasts^49^. Anthocyanin is responsible for the red/purple color of flowers, leaves, fruits and seeds. In strawberry, *RAP* (*Reduced Anthocyanin in Petioles*), a homolog of *AtGSTF12*, alters foliage and fruit color^50^. Here, we found that the TF genes *MYB5*, *MYB61*, *MYB118*, *MYB107*, and *TT2* (*MYB123*) were coexpressed with *BnGSTF6* and *BnGSTF12*. These TFs are responsible for seed coat development. *Medicago truncatula MYB5* mutants have darker seed coats than wild-type plants^51^. In *Arabidopsis*, TT2 is responsible for proanthocyanid in accumulation in developing seeds, as *TT2* mutant seed coats are golden yellow^52^. MYB118 represses endosperm maturation in *Arabidopsis* seeds^53^. MYB107 positively regulates suberin synthesis in the seed coat, as *myb107* seeds are darker than wild type^54^. The detailed roles of BnGSTF6, BnGSTF12, and the TFs coexpressed with these GSTs in *B*. *napus* should be elucidated in the future.

### GSTs plays pleiotropic roles in plants

In addition to its role in seed coat development, BnGSTF12 appears to play an important role in the response of *B*. *napus* to pathogen attack. *GSTF12* transcripts accumulate in *A*. *thaliana* in response to *Verticillium dahliae* infection^55^. GSTs play pleiotropic roles in improving plant tolerance to adverse environment conditions. Hodgkin^56^ defined seven types of pleiotropy and their underlying mechanisms, including artefactual pleiotropy, secondary pleiotropy, adoptive pleiotropy, parsimonious pleiotropy, opportunistic pleiotropy, combination pleiotropy and unifying pleiotropy. In poplar, alternative splicing at the *REVOLUTA* locus is responsible for the pleiotropic effects of this TF, which is associated with fungal resistance, leaf drop, and cellulose content^57^. In combination pleiotropy, a gene interacts with a variety of partners, which could alter its biochemical activity^56^. Gene duplication and the mutation of its regulatory partners play a leading role in pleiotropy^58^. In *Arabidopsis*, CBF1 regulates *UGT79B2* and *UGT79B3* in response to low temperatures, and other TFs might regulate *UGT79B2* in response to other environmental conditions^59^. In rice, the TF IDEAL PLANT ARCHITECTURE 1 (IPA1) enhances yield by increasing grains per panicle through activating the *DEP1* promoter and enhances immunity through binding to WRKY45^60,61^. Indeed, the promoter regions of the *BnGSTFs* include many *cis*-elements associated with abiotic and biotic stress responses. The pleiotropic roles of *BnGSTF12* in seed development and biotic stress tolerance might be regulated by different TFs. Due to the lack of an adequate number of samples, we were unable to perform coexpression analysis of genes involved in disease resistance.

### Expressional divergence of duplicate *GST* genes

Polyploidy and WGD are widespread in nature and are considered to be the main forces driving speciation and plant evolution. Most duplicated genes have arisen from WGD; studying the fate of duplicated genes is important for understanding plant evolution^62^. In the current study, 141 duplicate gene pairs were identified, including 10 containing pseudogenes. Duplicate genes produced by WGD are often lost or nonfunctional^63^. The Ka/Ks values of all duplicate genes except *BnaGST129* and *BnaGST139* were <1, indicating that the duplicate *BnaGST* genes have undergone extensive purifying selection. This idea is consistent with the finding that retained duplicate genes have experienced strong purifying selection^64,65^.

Functional and expressional divergence are important properties of retained duplicate genes. Indeed, 60.3% of full-length duplicated *BnGST* genes and most gene clusters showed divergent expression patterns, indicating that the duplicate gene pairs have undergone subfunctionalization or neofunctionalization. Expression bias or divergence has been observed in cotton (*Gossypium raimondii*), as approximately 93% of gene pairs are differentially expressed on a tissue basis^64^. Divergent expression between duplicates can arise due to the presence of different *cis*-regulatory elements^63^ or divergent microRNA binding sites^66^.

Epigenetic modification also has an important effect on expressional variation^67^. Structural divergence (differences in exon-intron structure) are prevalent in duplicate genes and can generate proteins with distinct biochemical functions^68^. A significant relationship between expressional and structural divergence has been observed in the bovine (*Bos taurus*) genome^69^. In the current study, 64.6% of duplicate gene pairs exhibited structural divergence, which might play an important role in the evolution of duplicate genes.

*GSTs* play multiple roles in plant development and stress responses. These genes could have important biotechnological applications through gene pyramiding and co-engineering^70^. A *GST* gene in maize was found to be associated with resistance to three plant diseases^71^. Ten *GST* genes were significantly upregulated after infection by *Botryosphaeria dothidea* in poplar^72^. Therefore, GSTs could be widely used for the improvement of disease resistance in plants. The overaccumulation of flavonoids can enhance tolerance to multiple stresses^73^: the *Arabidopsis ugt79b2/b3* double mutant exhibits reduced anthocyanin accumulation and increased sensitivity to stress^59^. Therefore, novel plant lines could be produced that overexpress *GST* genes. Perhaps novel crop varieties with enhanced biotic and abiotic stress tolerance could be generated through the overexpression of GSTs, likely leading to increased crop production.

## Main Conclusion

Coexpression network analysis of *GSTs* and genes encoding various transcription factors (NAC, MYB, WRKY and bZIP) points to different roles of GST during development in *B. napus*.

## Supplementary information

Supplementary Figure 1 Phylogenetic tree of A. thaliana (At), B. oleracea (Bo), B. rapa (Br), and B. napus (Bn) GST sequences

Supplementary Figure 2 Expression patterns of BnGST clusters in 21 different tissues at different developmental stages in B. napus

Supplementary Figure 3 Expression patterns of BnGSTs in response to Leptosphaeria maculans in B. napus

Supplementary Figure 4 Expression patterns of BnGSTs in response to Sclerotinia sclerotiorum in B. napus

Supplementary Table 1 GST protein sequences used to identify GST candidates in B. napus

Supplementary Table 2 GST protein identified in Brassica species

Supplementary Table 3 GST fragments identified in Brassica species

Supplementary Table 4 GST orthologous genes in A. thaliana, B. napus, B. oleracea and B.rapa

Supplementary Table 5 Gene duplication pattern in B. napus, B. oleracea and B. rapa

Supplementary Table 6 The Ka/Ks values, duplicaiton time, gene structures and expresion patterns for duplicated GST gene pairs in B. napus

Supplementary Table 7 Cis-elements of GSTs in B. napus

Supplementary Table 8 Coexpressed genes in various B. napus tissues
