# Supplementary material for: Genome wide identification and comparative analysis of glutathione transferases (GST) family genes in Brassica napus
Source: Sci Rep. 2019 Jun 24;9:9196. doi: 10.1038/s41598-019-45744-5 (PMC6591421; doi:10.1038/s41598-019-45744-5)
Supplement: Supplementary file 1 — Supplementary Figure 1 Phylogenetic tree of A. thaliana (At), B. oleracea (Bo), B. rapa (Br), and B. napus (Bn) GST sequences [file 41598_2019_45744_MOESM1_ESM.pdf]

Genome wide identification and comparative analysis of glutathione transferases (GST) family genes in Brassica napus

Lijuan Wei[1,2,†], Yan Zhu[1,2,†], Ruiying Liu [1,2,†], Aoxiang Zhang[1,2], Meicheng Zhu [1,2], Wen Xu[1,2], Ai Lin[1,2], Kun Lu[1,2], Jiana Li[1,2,*]

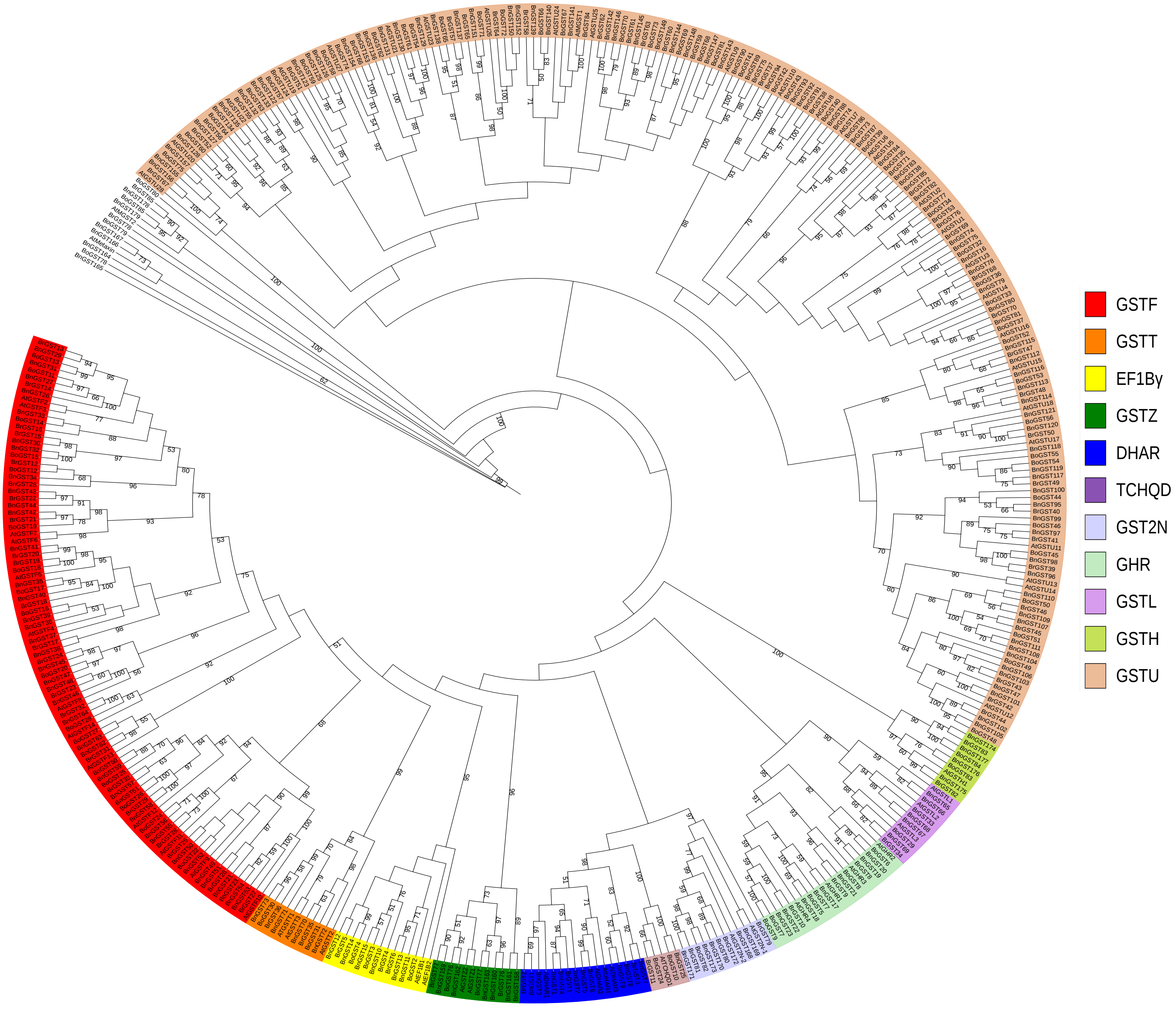

Supplementary Figure 1 Phylogenetic tree of *A. thaliana* (At), *B. oleracea* (Bo), *B. rapa* (Br), and *B. napus* (Bn) GST sequences.
