# Supplementary material for: Genome wide identification and comparative analysis of glutathione transferases (GST) family genes in Brassica napus
Source: Sci Rep. 2019 Jun 24;9:9196. doi: 10.1038/s41598-019-45744-5 (PMC6591421; doi:10.1038/s41598-019-45744-5)
Supplement: Supplementary file 3 — Supplementary Figure 3 Expression patterns of BnGSTs in response to Leptosphaeria maculans in B. napus [file 41598_2019_45744_MOESM3_ESM.pdf]

Genome wide identification and comparative analysis of glutathione transferases (GST) family genes in Brassica napus

Lijuan Wei[1,2,†], Yan Zhu[1,2,†], Ruiying Liu [1,2,†], Aoxiang Zhang[1,2], Meicheng Zhu [1,2], Wen Xu[1,2], Ai Lin[1,2],

Kun Lu[1,2], Jiana Li[1,2,*]

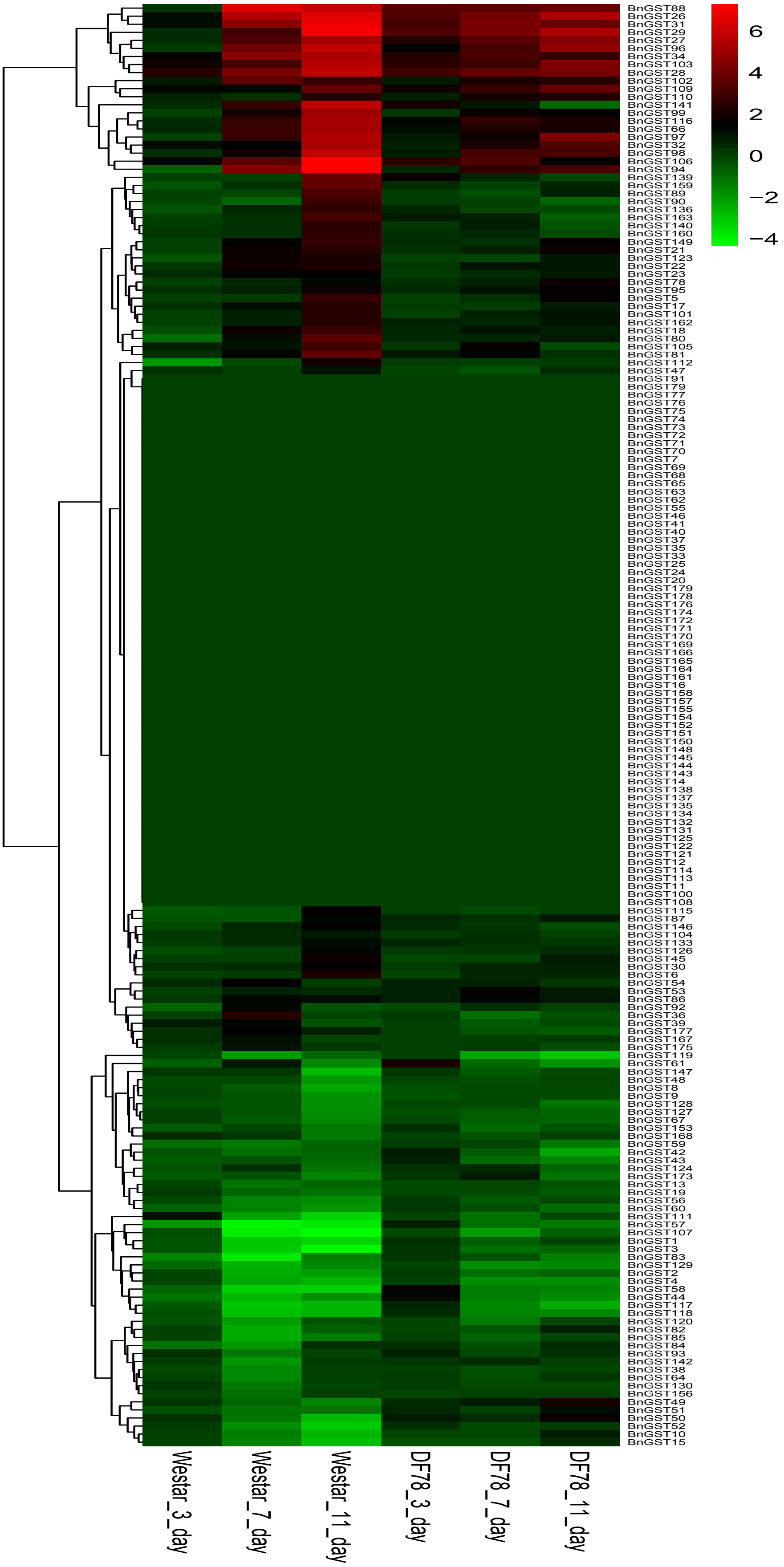

Supplementary Figure 3 Expression patterns of *BnGSTs* in response to *Leptosphaeria maculans* in *B. napus.*
